# Efficacy of Immunoglobulin Therapy for Secondary Prevention of Congenital Cytomegalovirus Infection: A Systematic Review and Meta-Analysis

**DOI:** 10.1093/ofid/ofag431

**Published:** 2026-07-16

**Authors:** Klaudia Gutowska, Anna Kucińska-Chahwan, Marta Bednarek, Anna Jelitto, Radosław Pietrzak, Patrycja Gumuła, Ranjit Akolekar, Tadeusz Issat

**Affiliations:** Department of Obstetrics and Gynecology, Institute of Mother and Child, Warsaw, Poland; Department of Obstetrics and Gynecology, Institute of Mother and Child, Warsaw, Poland; Department of Medical Genetics, Institute of Mother and Child, Warsaw, Poland; 2nd Department of Radiology, Medical University of Gdańsk, Gdańsk, Poland; Central Clinical Laboratory, University Clinical Center, Gdańsk, Poland; MRS Sp. z.o.o. (Ltd.), Warsaw, Poland; Department of Obstetrics and Gynecology, Institute of Mother and Child, Warsaw, Poland; Department of Obstetrics and Gynecology, Institute of Mother and Child, Warsaw, Poland; Medway Fetal and Maternal Medicine Centre, Medway NHS Foundation Trust, Gillingham, Kent, UK; Canterbury Christ Church University, Canterbury, UK; Department of Obstetrics and Gynecology, Institute of Mother and Child, Warsaw, Poland

**Keywords:** CMV, congenital, cytomegalovirus, hyperimmunoglobulin, pregnancy

## Abstract

Congenital cytomegalovirus (cCMV) is the leading infectious cause of long-term neuro-sensory impairment. Aim of meta-analysis was to evaluate the efficacy of antenatal immunoglobulin therapy—particularly cytomegalovirus-specific hyperimmune globulin (HIG)—in preventing vertical transmission and congenital cytomegalovirus infection (cCMV) in pregnancies complicated by primary maternal CMV infection. A search of PubMed, Cochrane Library, Embase, Scopus, ScienceDirect, Taylor & Francis Online, Wiley Online Library, ClinicalTrials.gov, and Google Scholar identified randomized controlled trials, prospective or retrospective cohort studies including pregnant women with serologically confirmed primary CMV infection. Eligible interventions included antenatal CMV-specific or nonspecific immunoglobulins (vs placebo, usual care, historical controls, or no treatment), although all included studies evaluated CMV-specific HIG. Controlled studies showed no significant reduction in transmission (RR 0.73, 95% CI .54–1.00; *P* = .051) with moderate heterogeneity. The pooled transmission rate after HIG was 27.2%, with substantial heterogeneity. Current evidence does not support routine antenatal immunoglobulin to prevent cCMV.

## Teaser

Meta-analysis of 13 studies shows antenatal CMV hyperimmune globulin does not significantly reduce congenital CMV transmission; evidence quality is low, and routine clinical use is not supported.

Primary cytomegalovirus (CMV) infection during pregnancy is substantially associated with major neurodevelopmental sequelae in affected fetuses, particularly when vertical transmission occurs in the first trimester. Prevention of vertical transmission and congenital CMV (cCMV) infection therefore represents a key clinical priority. One such potential strategy is the use of hyperimmune globulin (HIG) for the treatment and prevention of cCMV infection reported in a study in 1999 [1]. Over the last few decades, there have been many studies reporting their results investigating the role of HIG in preventing vertical transmission but there is contradictory evidence regarding its efficacy [2, 3]. Indeed, evidence suggests that the use of HIG may fail to reduce the rates of cCMV infection and may potentially increase the risk of other adverse pregnancy outcomes, including gestational hypertension, preeclampsia, small for gestational age, and perinatal death [2–4]. The contemporary use of HIG for prevention of cCMV infection is therefore empirical and lacks good quality evidence that needs to be investigated in robust studies.

## METHODS

### Eligibility Criteria

Study selection followed the Preferred Reporting Items for Systematic Reviews and Meta-Analyses (PRISMA 2020) guidelines [5]. Eligible studies investigated immunoglobulin therapy (CMV-HIG or nonspecific immunoglobulin preparations) for the prevention of maternal-fetal transmission of cytomegalovirus (CMV) in pregnant women with serologically confirmed primary CMV infection. Congenital infection was confirmed via polymerase chain reaction (PCR), viral culture, or antigenemia within three weeks postpartum.

Priority was given to randomized controlled trials, prospective or retrospective cohort studies, included in the meta-analysis published between 2015 and 2025. Comparators were placebo, usual care, historical controls, or no treatment. Single-arm cohorts were included for prevalence synthesis.

Studies were excluded if they focused on secondary CMV infection, viral reactivation, or immunocompromised populations. Research with missing data or published in languages other than English or Polish were excluded.

This systematic review was prospectively registered in the PROSPERO international register of systematic reviews (registration number: CRD420251131871).

### Information Sources and Study Selection

The search strategy was developed by an experienced systematic reviewer, in consultation with a medical librarian. Two reviewers independently screened all records.

Following the PRISMA 2020 guidelines [5], we conducted a comprehensive search across primary databases: PubMed, Cochrane Library, Embase, Scopus, ScienceDirect, Taylor & Francis Online, and Wiley Online Library. In addition, ClinicalTrials.gov was searched for clinical trials, and Google Scholar was used to identify studies not indexed in traditional databases. The complete search strategies are provided in Supplementary 1. The PRISMA 2020 checklist is provided in Supplementary 4. The MOOSE checklist is provided in Supplementary 5.

The initial search identified 4727 records. Two independent reviewers screened titles and abstracts, followed by full-text assessment of potentially eligible articles using Zotero software (v7.0). Of 26 reports assessed for eligibility, 13 were excluded, and 13 studies met the inclusion criteria (six controlled and seven observational). The excluded studies and the reasons for exclusion are detailed in Supplementary 2. Both controlled trials and observational studies were eligible; the inclusion of observational studies was justified by the practical and ethical constraints that limit the feasibility of conducting large placebo-controlled trials in pregnant women with active CMV infection. Discrepancies were resolved by discussion and consensus. Data extraction and risk-of-bias assessment were performed independently by the same reviewers using standardized forms. The 13 included studies were analyzed quantitatively in the meta-analysis and qualitatively in the systematic synthesis.

### Data Extraction

A standardized data extraction form was developed in accordance with the Cochrane Handbook for Systematic Reviews of Interventions [6]. Two reviewers independently extracted data using piloted forms, and the results were transcribed into a dedicated database.

Extracted information included key characteristics (year of publication, study location, design, clinical setting, and sample size), participant demographics, baseline characteristics, inclusion and exclusion criteria applied in each study. Intervention details included the type of immunoglobulin administered, dosing regimen, timing, and duration of treatment.

Primary outcome data comprised the number of participants in each study arm, the number of cCMV infections, and effect estimates with corresponding confidence intervals, when available. Secondary outcomes and subgroup analyses were extracted when reported. All extracted data were cross verified for accuracy and completeness. When necessary, corresponding authors were contacted to clarify ambiguous information or to obtain additional unpublished data.

### Risk-of-bias Assessment

Two reviewers independently assessed the risk of bias for included studies. Randomized controlled trials were evaluated using the Cochrane Risk of Bias 2 (RoB 2) tool [7], which assesses five domains. Nonrandomized studies were evaluated using the Risk of Bias In Nonrandomized Studies-of Interventions (ROBINS-I) tool [8], covering seven domains from confounding to selection of the reported result.

Publication bias was evaluated through visual inspection of funnel plots and statistical testing using Egger's regression test for funnel plot asymmetry. These analyses provided an indication of the potential influence of publication bias on effect estimates and the robustness of the meta-analysis findings.

### Statistical Analysis

All analyses were conducted in R version 4.5.0 using the meta (v8.1.0), metafor (v4.8.0), esc (v0.5.1), robumeta (v2.1), and robvis (v0.3.0) packages. Two meta-analyses were performed to address research questions and account for heterogeneity in study design.

The first meta-analysis included controlled studies (n = 6) comparing immunoglobulin therapy with a control group [3, 9–13]. Risk ratios (RRs) with 95% confidence intervals (CIs) were calculated for binary outcomes using the Mantel-Haenszel method. A random-effects model was fitted with the Paule-Mandel (PM) estimator for between-study variance (*τ*^2^), and the Hartung-Knapp adjustment was applied to account for uncertainty in *τ*^2^ estimation [14].

The second meta-analysis included 13 studies to estimate the overall prevalence of cCMV infection following immunoglobulin therapy. Proportions were analyzed using logit transformation to stabilize variance. Study-specific CIs were calculated using the Clopper-Pearson method.

Heterogeneity was evaluated using Cochran's Q-test, the *I*^2^ statistic with 95% uncertainty intervals, and *τ*^2^ estimates derived from PM method, with CIs for *τ*^2^ obtained via the Q-profile method. Prediction intervals were calculated for random-effects models to indicate the expected range of effects in future studies. Sensitivity analyses assessed the robustness of results by excluding studies at high risk of bias and conducting leave-one-out influence analyses. Publication bias was examined via funnel plot inspection and Egger's regression test for funnel plot asymmetry, with sensitivity checks for selective reporting and small-study effects. All effect estimates are reported with 95% CIs, and statistical significance was set at *P* < .05. Forest and funnel plots were generated to visually present study-specific results and pooled estimates.

### Assessment of Certainty of Evidence

We assessed the certainty of evidence for the primary outcome using the Grading of Recommendations Assessment, Development and Evaluation (GRADE) approach. The complete GRADE evidence profile is provided in Supplementary 3.

## RESULTS

### Search Results

The systematic search identified 4727 records. After removing 82 duplicates and excluding 4188 clearly irrelevant records, 457 unique records remained for title and abstract screening. Of these, 431 were excluded at the title/abstract stage primarily because they did not involve CMV immunoglobulin therapy in pregnant women or did not report congenital transmission as an outcome. The remaining 26 reports proceeded to full-text review; all were successfully retrieved.

**Figure 1.**
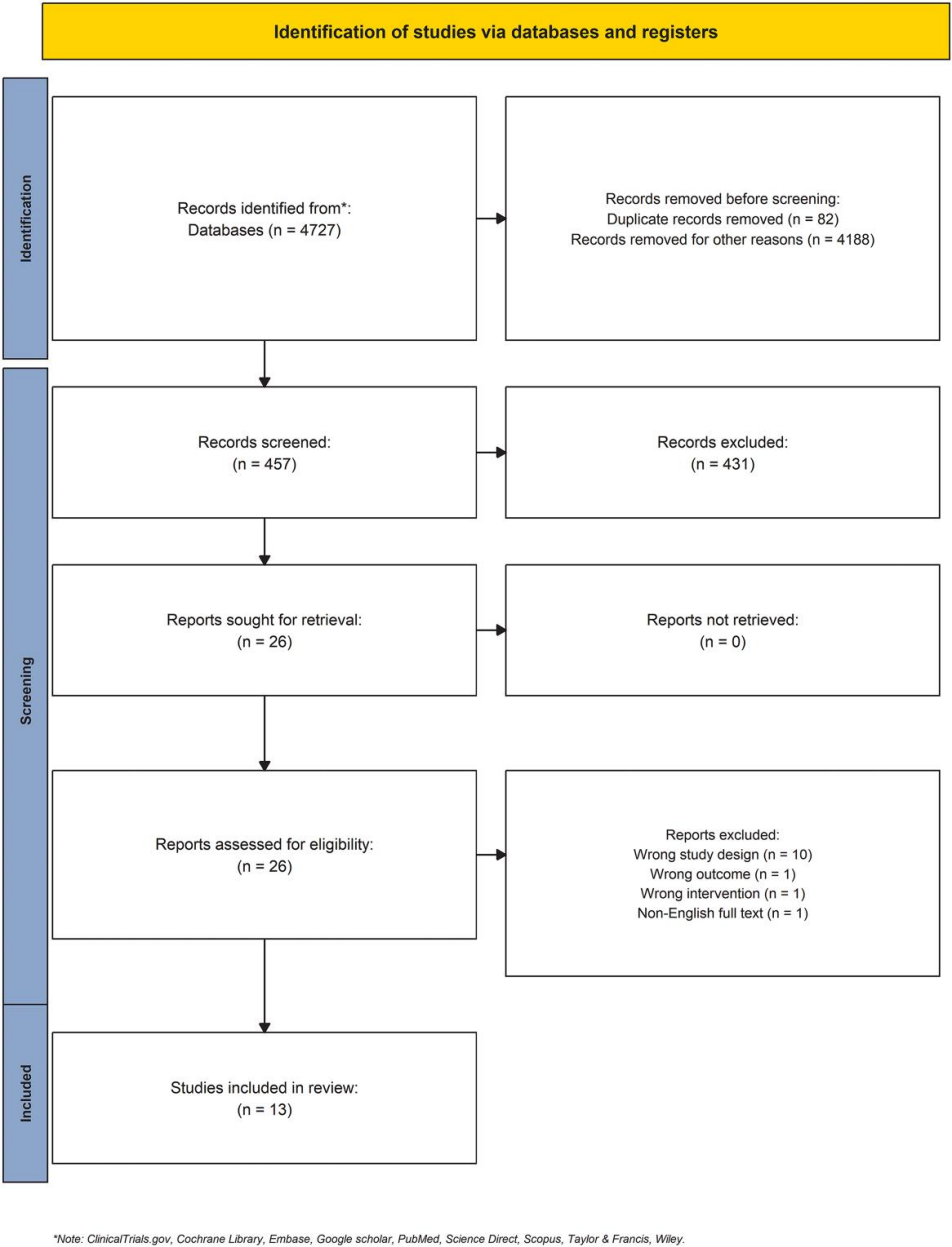
Study selection process (PRISMA 2020 flowchart).

Following detailed assessment, 13 reports were excluded: ten for ineligible study design, one for reporting the wrong outcome, one for using the wrong intervention, and one due to the full text not being available in English. The remaining 13 studies met the predefined eligibility criteria and were included in the review (Figure 1).

### Study Characteristics

The main characteristics of the 13 studies included in the systematic review and meta-analysis are presented in Table 1. There were 2 randomized controlled studies [3, 9], 3 prospective cohort studies [15–17] and 8 retrospective cohort studies [10–13, 18–21]. The sample size varied considerably between studies ranging from 5 to 162 in retrospective cohort studies, 40 to 149 in prospective studies and 45 to 206 in randomized studies. All studies evaluated CMV-specific HIG administered to pregnant women with primary CMV infection. Control groups consisted of no treatment, usual care, or placebo. Overall risk of bias ranged from moderate to serious, with the majority of studies rated as having a serious risk of bias. Regarding diagnostic methods for confirming congenital infection, PCR-based CMV DNA detection in amniotic fluid and/or neonatal urine or saliva was the primary method in 12 of 13 studies (n = 978 participants in the prevention group), including both RCTs and the majority of cohort studies. Two of those 12 studies (Delle Chiaie et al [18], n = 50; Minsart et al [10], n = 5) used PCR in combination with viral culture for at least one specimen type; given the substantially lower sensitivity of culture compared with PCR, any culture-positive results in these studies should be interpreted in that context. One study (Seidel et al [17], n = 46) reported CMV detection without specifying the methodology. Serological confirmation of primary maternal infection was based predominantly on IgG seroconversion (absence of CMV IgG at an earlier date followed by appearance) or on a combination of CMV IgM positivity and low IgG avidity index. No study relied on a four-fold IgG titer rise alone. The heterogeneity in diagnostic methodology—particularly between PCR and culture, and between seroconversion and IgM/avidity approaches—may affect the sensitivity of outcome ascertainment and the comparability of transmission rates across studies.

**Table 1.** Study Design and Participants Characteristics

First Author	Year	Time Set	Study Design	Country	Intervention	PG (m/f)	CG (m/f)	GA At Dx (wks)	GA At HIG Init (wks)	HIG Regimen	Dosing Interval	Method	Result (cCMV)
Devlieger et al [9]	2021	2008–2016	RCT—open-label	Multinational	CMV-HIG	45/45	34/28	PG: Mdn 24 [11–35]	PG: Mdn 24 [11–35]	200 IU/kg	q2w → q4w	AF: PCR; NB: Urine/Blood	No benefit (open-label RCT; no sig. reduction in transmission)
Hughes et al [3]	2021	2012–2018	RCT—double-blind	USA	CMV-HIG	206/203	191/191	PG: M 16.2 ± 3.9; CG: 15.6 ± 4.1	Same as dx^a^	100 mg/kg	q4w	AF: PCR.; NB: Sal./Uri.	No benefit (double-blind RCT; no sig. reduction; *P* > 0.05)
Kagan et al [15]	2019	2013–2017	PC	DE, BE	CMV-HIG	40/40	N/A	T1, Mdn 9.6 [7.5–11.4]	Mdn 11.1 [8.5–12.4]	200 IU/kg (mod.)	q2w	AF: PCR; NB: Uri./Sal.	No control group; 6/40 (15%) transmission
Kagan et al [16]	2021	2013–2020	PC	Germany	CMV-HIG	149/153	N/A	T1, Mdn 9.6 [8.1–11.4]	Mdn 10.6 [9.1–12.3]	200 IU/kg (mod.)	q2w	AF: PCR; NB: Uri./Sal.	No control group; lowest rate (6.5%) in pooled analysis
Seidel et al [17]	2020	2010–2017	PC	Germany	CMV-HIG	46/46	281/281	PG: Mdn 14.0 [5.0–31.0]	PG: Mdn 17.0 [6.0–34.0]	200 IU/kg	q2w	AF: CMV detect; NB: Urine	No benefit (controlled; no sig. reduction in transmission vs historical CG)
Nigro et al 2015 [11]	2015	2005–2013	RC	IT/USA	CMV-HIG	162/162	194/194	PG: M 15.8 ± 7.5	PG: M 22.4 ± 6.1	100–200 IU/kg or mg/kg	q4w	AF: PCR (CMV DNA); NB: N/A	Potential benefit (controlled; reduced transmission in PG vs CG)
Delle Chiaie et al [18]	2018	2007–2016	RC	Germany	CMV-HIG	50/49	N/A	Periconc.−T3, Mdn 13 [8.3–21]	+2 w from dx	200 IU/kg	q2–3w	AF: PCR/cult.; NB: Urine	No benefit (no control group; single-arm)
Minsart et al [10]	2018	2005–2016	RC	Canada	CMV-HIG	5/5	26	T1–T3 (81.3% <20 w)	[no data]	150 mg/kg	q4w	AF: PCR; NB: PCR/cult.	No benefit (controlled; 1/5 PG vs 10/26 CG; RR 0.52; very small n)
Blázquez-Gamero et al [19]	2019	2009–2015	RC	Spain	CMV-HIG	17/17	N/A	T1–T3, Mdn 20 [10–25]	Mdn 24 [19.5–28]	100 or 200 IU/kg	q4w	AF: PCR; NB: Urine	No benefit (no control group; 7/17 = 41% transmission)
Nigro et al 2020 [12]	2020	2010–2017	RC	IT/USA	CMV-HIG	157/157	148/147	PG: M 13.6 ± 7.9; CG: 11.7 ± 7.3	PG: M 22.0 ± 7.0; CG: 19.1 ± 6.8	200 IU/kg	q3w	AF: PCR; NB: PCR	Potential benefit (controlled; lower transmission with high-dose HIG)
Richtmann et al [13]	2022	2014–2020	RC	Brazil	CMV-HIG	6/6	13/13	Mdn 17.3 [6–38]	N/A (Pre-amnio)	70 IU/kg	q4w	AF: PCR; NB: Urine	No benefit (controlled; 0/6 PG vs 1/13 CG, very small n)
Schirwani-Hartl et al [20]	2023	2010–2022	RC	Austria	CMV-HIG	36/34	N/A	T1-T2, Mdn 11.6 [8.6–14.0]	Mdn 13.1 [11.9–17.0]	200–300 IU/kg	q4w/q2w	AF: PCR; NB: Urine	No control group; 7/36 (19%) transmission
Karofylakis et al [21]	2024	2009–2022	RC	Greece	CMV-HIG	107/107	N/A	T1, Mdn 9 [8–11]	Mdn 13 [10–15]	200 IU/kg (mod.)	q4w/q2w	AF: PCR; NB: Urine	No control group; 32/107 (30%) transmission

Abbreviations: AF, amniotic fluid; CG, control group; cult., viral culture; Dx, diagnosis; GA, gestational age; HIG, hyperimmune globulin; IU/kg, international units per kilogram; M, Mean ± Standard Deviation; m/f, number of mothers/number of fetuses; Mdn, median; mod., modified high-dose regimen; N/A, not available; NB, newborn; PC, prospective cohort; PCR, polymerase chain reaction; Periconc., periconceptional; PG, prevention group; Pre-amnio, Preamniocentesis; q2w, every 2 weeks; q3w, every 3 weeks; q4w, every 4 weeks; RC, retrospective cohort; RCT, randomized controlled trial; Sal., saliva; T1–T3, first to third trimester; U/kg, units per kilogram; Uri., urine; w/wks, weeks.

^a^In Hughes et al (2021), gestational age at diagnosis corresponds to gestational age at randomization and treatment initiation; screening occurred on average approximately 3–4 weeks earlier.

### Risk-of-bias in Studies

The 11 nonrandomized cohort studies were evaluated with ROBINS-I [8] across seven domains: D1 (bias due to confounding), D2 (bias in selection of participants), D3 (bias in classification of interventions), D4 (bias due to deviations from intended interventions), D5 (bias due to missing data), D6 (bias in measurement of outcomes), and D7 (bias in selection of the reported result).

**Figure 2.**
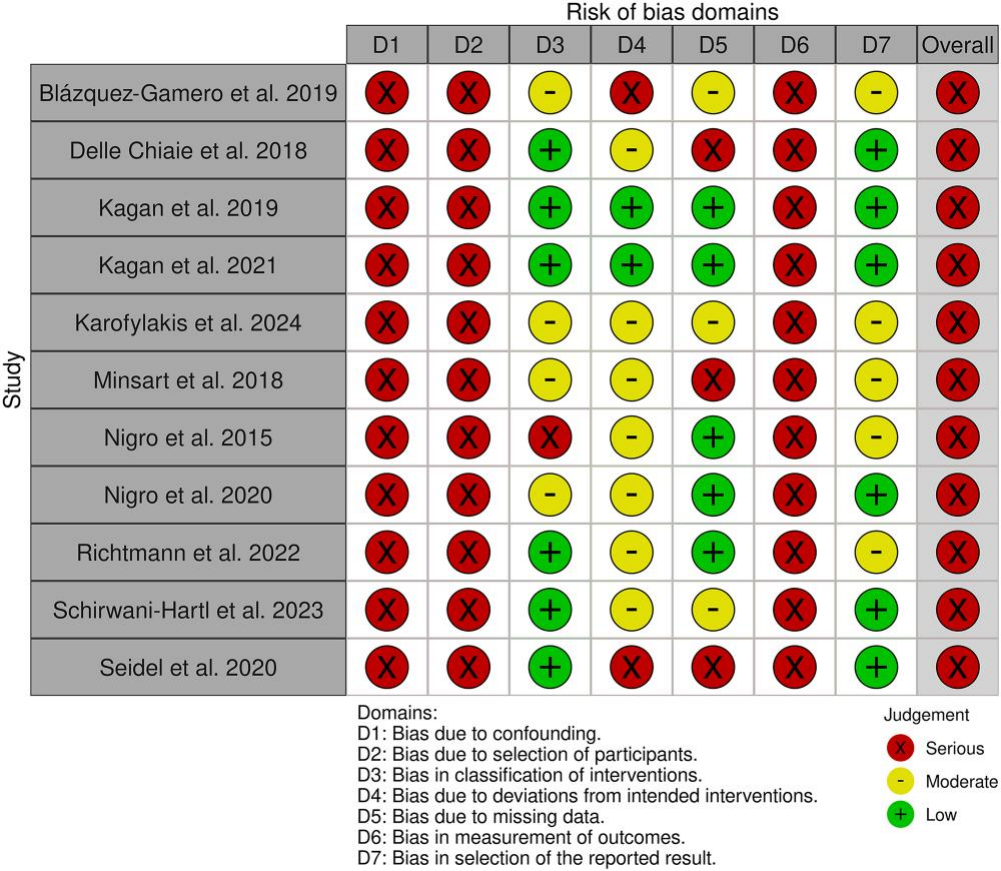
Risk-of-bias assessment of included studies using the ROBINS-I tool.

As shown in Figure 2, most studies had serious risk of bias, particularly confounding, participant selection, deviations from intended interventions, and measurement of outcomes. Low-risk ratings were mostly limited to classification of interventions and selection of reported results.

**Figure 3.**
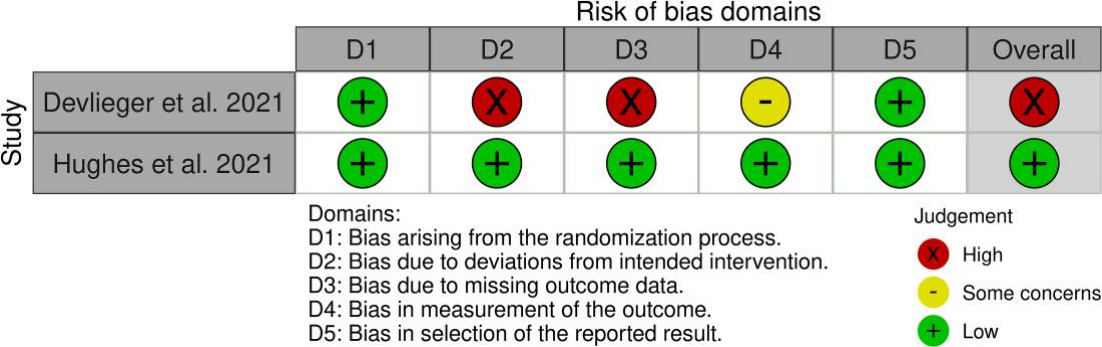
Risk-of-bias assessment of included RCT studies using the RoB 2 tool.

The two randomized trials, Devlieger et al [9] and Hughes et al [3], were evaluated with the Cochrane RoB 2 tool [7] for the primary outcome (Figure 3). Hughes et al [3] was judged to be at low risk of bias across all domains, reflecting a centralized randomization procedure, double blinding with a credible placebo, missing outcome data (note: the GRADE evidence profile flags Hughes et al [3] as having >20% missing outcome data, warranting caution in risk-of-bias interpretation for this domain), blinded outcome assessment, and prespecified analyses.

In contrast, Devlieger et al [9] was judged to be at high risk of bias overall. This rating was driven by a high risk of bias due to deviations from intended interventions (D2), attributed to the open-label design and off-label HIG administration in the control arm, and a high risk of bias due to missing outcome data (D3) among seroconverted participants. Additionally, the measurement of the outcome (D4) raised some concerns given the lack of blinding and potential for differential screening intensity.

All cohort studies were rated as serious risk overall. For example, Nigro et al [11, 12] and Kagan et al [15, 16] had serious concerns in multiple domains, reflecting potential selection bias and lack of control for confounding factors. Smaller single-center cohorts, such as those by Minsart et al [10] and Richtmann et al [13], demonstrated serious risks related to incomplete outcome data and deviations from intended protocols.

The predominance of serious risk ratings underscores limitations in the methodological rigor of most included studies. From a GRADE perspective, this body of evidence would be downgraded for risk of bias, contributing to an overall low to very low certainty rating for key outcomes. This high prevalence of bias reduces confidence in effect estimates and highlights the need for future well-designed randomized controlled trials.

### Meta-analysis: Prevention Versus Treatment

A meta-analysis of six controlled studies [3, 9–13] compared HIG therapy with control groups for the prevention of cCMV infection. The analysis included 1177 participants (578 in treatment groups and 599 in control groups), with 525 cCMV infection events recorded across all studies.

**Figure 4.**
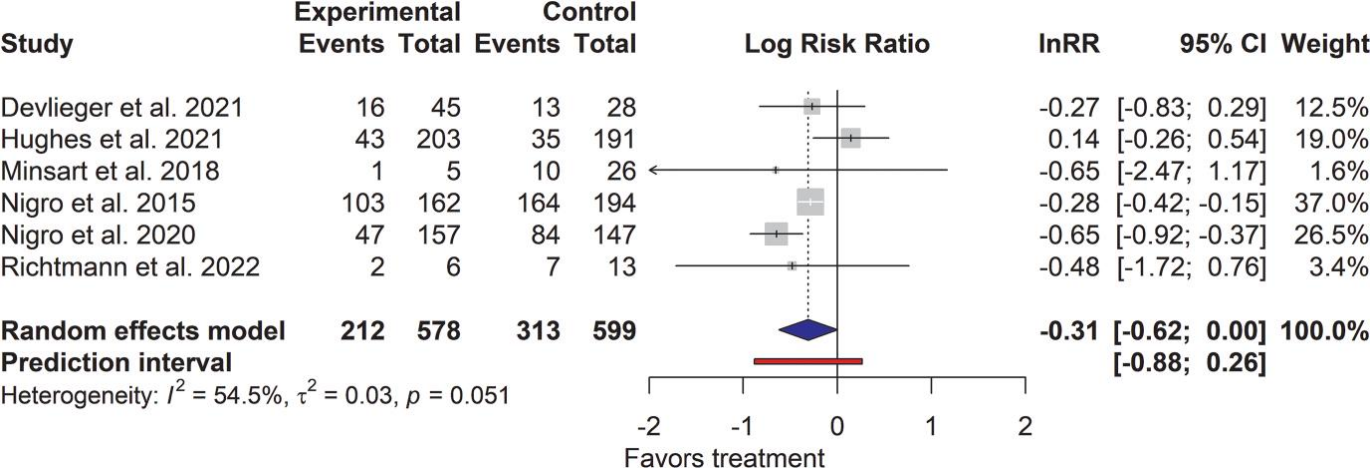
Forest plot of log risk ratios for congenital CMV infection.

The random-effects model (Hartung-Knapp adjustment applied) yielded a pooled RR of 0.73 (95% CI: .54–1.00], *P* = .051), corresponding to a log RR of −0.31 (Figure 4). While this point estimate suggests a potential 27% relative reduction in infection risk, the confidence interval crossed 1.0, indicating no statistically significant effect and substantial uncertainty about the presence of any true benefit.

Moderate heterogeneity was observed between studies (*I*^2^ = 54.5%, 95% CI: 0.0%–81.8%, *τ*^2^ = 0.035, *Q* = 11.00, df = 5, *P* = .051). The Q-test approached statistical significance, suggesting some variation in treatment effects between studies. The prediction interval was wide, reflecting uncertainty in the range of true effects that could be expected in future research.

#### Sensitivity Analysis

To evaluate the robustness of the primary pooled estimate (RR 0.73, 95% CI .54–1.00), two complementary sensitivity analyses were conducted.

First, a leave-one-out influence analysis was performed under the same random-effects (PM/Knapp-Hartung) framework. Omitting Devlieger et al [9], Minsart et al [10], Nigro et al [11], or Richtmann et al [13] resulted in minimal changes to the pooled RR (0.73–0.74) and only modest variations in *τ*^2^ (0.049–0.052) and *I*^2^ (61–64%). In contrast, excluding Hughes et al [4] reduced the RR to 0.68 (95% CI: .55–0.85]), *τ*^2^ to 0.005, and *I*^2^ to 28%. Excluding Nigro et al [12] yielded an RR of 0.79 (95% CI: .64–.97]), *τ*^2^ = 0.002, and I^2^ = 8%. These results indicate that Hughes et al [3] and Nigro et al [12] had the greatest influence on both effect size and heterogeneity.

Second, a trimmed random-effects model was fitted with both influential studies excluded, removing 479 participants (316 events). This analysis produced an RR of 0.75 (95% CI: .71–0.80]), eliminated between-study heterogeneity (*τ*^2^ = 0 [0.000, 0.056]; *I*^2^ = 0%, 95% CI: 0%–84.7%]), and yielded a statistically significant pooled effect (*P* < .001). However, this “best-case” scenario excluded 57.5% of participants and 37.7% of events from the prevention group and lacked any predefined methodological rationale. Therefore, all six controlled studies were retained in the primary analysis, with the trimmed model reported solely as a robustness check.

#### Publication Bias

**Figure 5.**
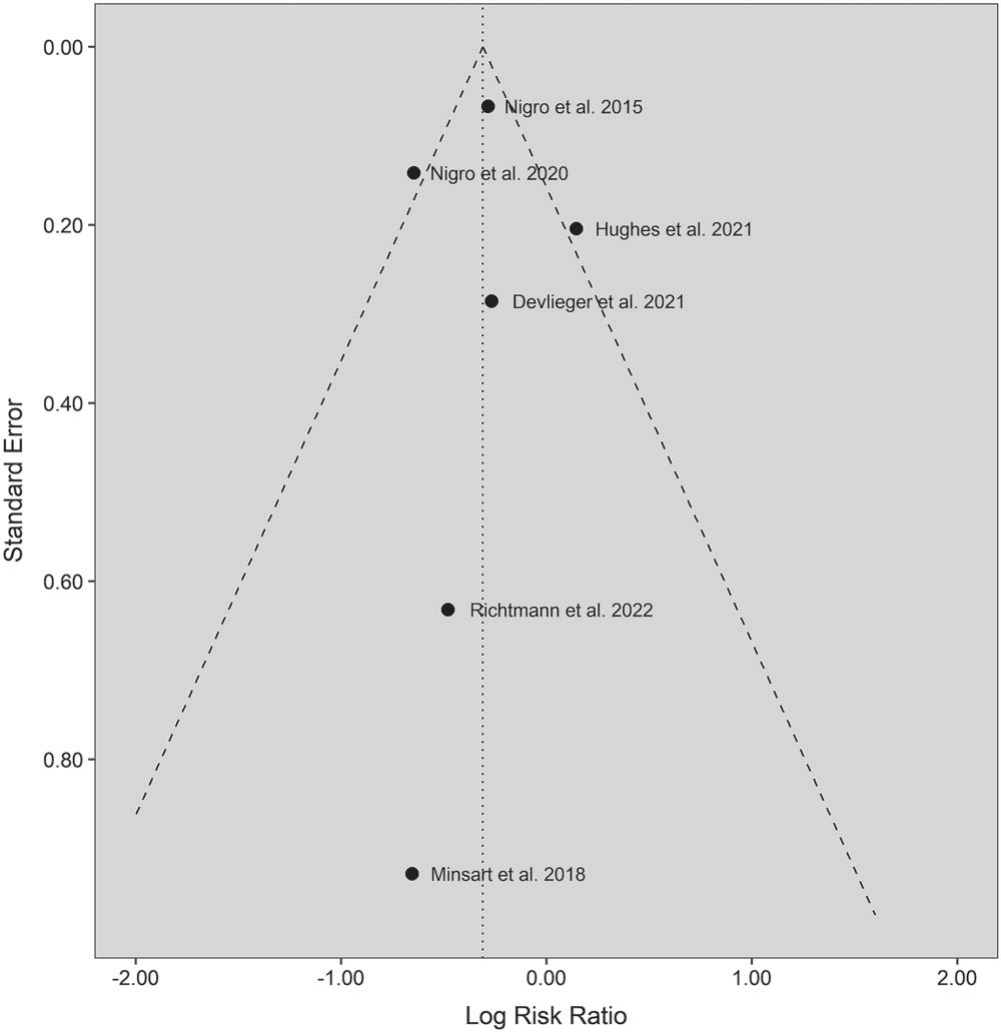
Funnel plot for the meta-analysis of controlled studies.

The funnel plot of the six controlled studies (Figure 5) appeared symmetric around the pooled log RR, with no evident gaps in the region of highest precision. Egger's regression test yielded t = −0.08 (df = 4, *P* = .943), and Begg's rank correlation was also nonsignificant. Given the low number of studies, both tests are underpowered, and results should be interpreted cautiously.

To complement these analyses, a weight-function model was applied. The adjusted log RR (−0.29, 95% CI: −0.58, 0.00]) was virtually identical to the unadjusted estimate, and the likelihood ratio test indicated no evidence of selective reporting (*P* = .698). We found no indication of small-study effects or publication bias; however, uncertainty remains due to the limited number of studies.

### Overall Congenital Cytomegalovirus Transmission Rate Following Hyperimmune Globulin Therapy

**Figure 6.**
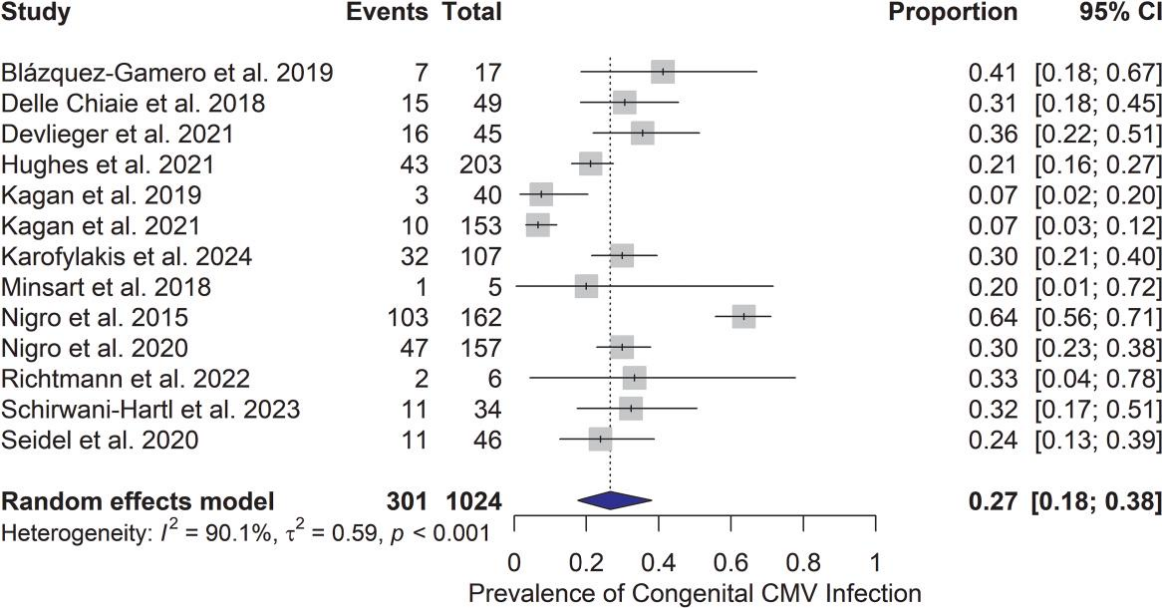
Forest plot of pooled cCMV transmission rates across all 13 studies (proportions analysis).

This pooled proportion analysis (Figure 6) included all 13 studies (controlled and observational) to estimate the rate of cCMV transmission in pregnant women receiving immunoglobulin therapy. Across 1024 participants, 301 infection events were reported.

The random-effects model estimated a pooled transmission rate of 27.2% (95% CI: 18.8%–37.7%), indicating that approximately one in four pregnancies with primary maternal CMV infection resulted in congenital transmission despite HIG administration.

Heterogeneity was very high (*I*^2^ = 90.1%, 95% CI: 84.9%–93.5%; *τ*^2^ = 0.61; *Q* = 120.82, df = 12, *P* < .001), suggesting substantial variation in observed transmission rates. Individual study estimates ranged from 6.5% [16] to 63.6% [11], reflecting differences in patient populations, intervention timing, dosing regimens, and methodological approaches. The magnitude of heterogeneity indicates that much of the variation between studies likely reflects true differences in effect rather than sampling error, warranting cautious interpretation of the pooled estimate.

#### Sensitivity Analysis

The influence analysis identified two studies with a substantial impact on the pooled estimate and heterogeneity. Omitting Nigro et al [11] produced the largest changes, reducing the pooled proportion from 27.2% to 24.3% and heterogeneity from *I*^2^ = 90.1% to 72.7% (*τ*^2^ = 0.61 to 0.34). Excluding Kagan et al [16] also lowered heterogeneity (*I*^2^ = 87.0%) while slightly increasing the pooled proportion to 30.8%. Removing any other single study produced only minor changes in the pooled estimate (range: 26.2%–29.3%), with heterogeneity consistently remaining very high (*I*^2^ > 90%).

A trimmed analysis excluding both Nigro et al [11] and Kagan et al [16] included 11 studies with 709 participants and 188 cCMV infection events-representing 70% of the original sample and 62% of observed events. The pooled transmission rate was 27.6% (95% CI: 23.5%–32.2%), but heterogeneity was markedly reduced (*I*^2^ = 35.7%, 95% CI: 0%–68.4%; *τ*^2^ = 0.033). The Q-test for heterogeneity was no longer statistically significant (*Q* = 15.56, df = 10, *P* = .113).

Given the absence of methodological justification for excluding these studies and the importance of capturing the full range of transmission rates in the literature, both influential studies were retained in the primary analysis. The trimmed model is reported only as a robustness check to illustrate their influence on heterogeneity.

#### Publication Bias

Egger's regression test indicated no evidence of funnel plot asymmetry (*t* = −1.12, df = 11, *P* = .287), with a bias estimate of −2.14 (SE = 1.91). The nonsignificant result suggests no systematic association between study precision and effect size that would indicate publication bias or small-study effects.

The weight-function model incorporated a multiplicative residual heterogeneity variance (*τ*^2^ = 9.86); this value is specific to that sensitivity model and is distinct from the *τ*^2^ = 0.61 estimated in the primary random-effects proportions model.

## DISCUSSION

### Principal Findings

The principal findings of our study are that firstly, there is considerable heterogeneity in studies reporting the use of HIG therapy in pregnancies with CMV infection with majority of included studies demonstrating serious bias; secondly there is no significant reduction in the rate of cCMV infection in pregnancies that were treated with HIG compared with those that were not, lastly, there is no robust evidence of efficacy in reducing rate of cCMV infection in pregnancies treated with HIG.

This systematic review and meta-analysis evaluated the efficacy of immunoglobulin therapy-primarily CMV-specific HIG for the prevention of cCMV infection in pregnant women with primary CMV infection. The analysis encompassed over 1000 participants. In controlled studies, HIG administration was associated with a nonsignificant reduction in the risk of cCMV infection. Although the point estimate suggests a potential reduction of approximately 27%, the confidence interval included the null value, indicating that these findings do not provide robust evidence of efficacy. The prediction interval further highlighted the uncertainty surrounding the true effect in future populations.

### Comparison With Existing Literature

The absence of a statistically significant effect in controlled studies aligns with trials such as Hughes et al [3] reporting no benefit over placebo. Earlier uncontrolled studies and retrospective analyses suggested more favorable outcomes, but are prone to bias from confounding, patient selection, and variable diagnostic protocols. Potential explanations for the inconsistent results include the timing of intervention. Additionally, variations in dosing regimen and population heterogeneity, such as variations in maternal age, immune status, baseline viral load, and comorbidities, may influence treatment responsiveness. Finally, methods discrepancies, including the use of PCR, viral culture, or antigenemia assays, could contribute to outcome variability. Our findings are broadly consistent with the 2021 systematic review and meta-analysis by El-Qushayri et al [22] which searched nine databases, included eight studies, reported a pooled cCMV prevalence of 36.5% (95% CI 26%–49%), and found no evidence that HIG is effective against congenital CMV (OR 0.53, 95% CI 0.20–1.42). Key differences between that review and the present analysis include: (i) we include 13 studies (vs 8), capturing more recent and larger trials (Hughes et al [3]; Devlieger et al [9]) that substantially increase total participant numbers; (ii) we restrict our date range to 2015–2025 to reflect contemporary diagnostic standards (PCR-confirmed cCMV) and modern HIG formulations; and (iii) we separately analyze controlled studies and single-arm cohorts, yielding both a relative risk estimate and a pooled prevalence. A consequence of the 2015 start date is that the pivotal RCT by Revello et al published in the New England Journal of Medicine [2] was not included in the quantitative analysis. However, the Revello trial's finding—no significant reduction in congenital transmission with CMV-HIG—is directionally concordant with the pooled result reported here. Future updates should consider extending the search to pre-2015 studies to fully capture the evidence base.

### Heterogeneity and Methodological Considerations

Both the RR and prevalence meta-analyses demonstrated between-study heterogeneity, particularly in the prevalence analysis. Sensitivity analyses revealed that individual influential studies [11, 16] accounted for a considerable proportion of this variability, but exclusion of these studies did not meaningfully change the pooled prevalence. This suggests that high heterogeneity reflects differences in study populations, intervention protocols, and methodological quality rather than statistical anomalies.

Risk-of-bias assessment using the ROBINS-I tool revealed that most observational studies were at serious risk of bias. Funnel plot inspection and Egger's tests for both the RR and prevalence analyses did not identify significant publication bias. However, the small number of controlled studies limits the statistical power of these tests. It is also noteworthy that the pooled RR in controlled studies (RR 0.73, 95% CI .54–1.00; *P* = .051) approached but did not reach statistical significance at the conventional 0.05 threshold. The point estimate suggesting a 27% relative risk reduction, combined with a confidence interval narrowly crossing the null, reflects a biologically plausible trend that warrants acknowledgment. These findings should not be interpreted as ruling out a clinically meaningful benefit, particularly given the limited power of the available RCTs. However, in the absence of statistical significance and given the broad prediction interval, current evidence is insufficient to support routine use of HIG. Regarding safety, it is important to acknowledge that prenatal complications associated with CMV-HIG have been reported. Hughes et al [3] observed a higher rate of preterm birth in the HIG arm compared with placebo, and the trial by Revello et al [2] identified a trend toward increased obstetric complications including preterm labor and preeclampsia in treated women. These findings raise important safety considerations that must be factored into clinical decision-making, particularly given the lack of demonstrated efficacy. The potential for harm reinforces the conclusion that HIG should not be administered outside of carefully monitored research protocols.

### Strengths

Our systematic review has several methodological strengths. We conducted a comprehensive search across 9 databases. Dual independent screening, data extraction, and risk-of-bias assessment minimized subjective bias. We performed multiple prespecified sensitivity analyses that strengthened confidence in our findings. The separate analysis of controlled studies and single-arm cohorts provided complementary perspectives on both comparative effectiveness and real-world treatment outcomes. Finally, we assessed publication bias using multiple methods and employed GRADE methodology to provide transparent quality-of-evidence ratings.

### Limitations

Several important limitations must be acknowledged. First, despite the lack of a statistically significant finding in controlled studies, the overall certainty of evidence is low to very low by GRADE criteria. Second, substantial heterogeneity characterized the intervention protocols across studies. Timing of hyperimmunoglobulin administration relative to maternal infection was highly variable and often poorly documented. Third, outcome definitions varied across studies. Some studies assessed congenital infection at amniocentesis, while others used infection status at birth. We extracted outcome each study reported as primary, introducing potential heterogeneity. Fourth, we did not have access to individual-level data on the precise timing of maternal seroconversion or interval between infection and treatment initiation for most studies. Finally, most studies did not report or adequately control for important potential confounders including maternal immune status, baseline viral load, obstetric history, or socioeconomic factors that might influence both treatment allocation and outcomes.

### Conclusions

At present, the evidence does not support routine use of HIG for prevention of congenital CMV infection in pregnant women with primary infection outside of research settings. While observational data have suggested potential benefit, the most rigorous trials to date have failed to demonstrate a statistically significant effect, and residual uncertainty remains due to heterogeneity and methodological limitations. Clinicians should weigh potential benefits against cost, limited availability, and the absence of high-certainty evidence before recommending HIG prophylaxis.

## Supplementary Material

ofag431_Supplementary_Data

## Supplementary Data

Supplementary materials are available at *Open Forum Infectious Diseases* online. Consisting of data provided by the authors to benefit the reader, the posted materials are not copyedited and are the sole responsibility of the authors, so questions or comments should be addressed to the corresponding author.
