# Supplementary material for: Efficacy of Immunoglobulin Therapy for Secondary Prevention of Congenital Cytomegalovirus Infection: A Systematic Review and Meta-Analysis
Source: Open Forum Infect Dis. 2026 Jul 16;13(7):ofag431. doi: 10.1093/ofid/ofag431 (PMC13397120; doi:10.1093/ofid/ofag431)
Supplement: ofag431_Supplementary_Data [file ofag431_supplementary_data.zip › Supplement_1_Database_and_Registry_Search_Strategies.docx]

# Supplement 1. Complete database and registry search strategies

**Review title:** *Efficacy of immunoglobulin therapy for secondary prevention of congenital cytomegalovirus infection: a systematic review and meta-analysis*

**PROSPERO registration:** CRD420251131871

**Search period:** 2015-01-01 to 2025-06-15
**Language limits:** English and Polish
**Last comprehensive search:** 2025-06-15 (Europe/Warsaw, CEST)
**Deduplication software:** Zotero 7.0 (Corporation for Digital Scholarship)

## eTable S1A. Source attributes and search log

| No. | Database/Register | Date Searched | Records Retrieved |
| --- | --- | --- | --- |
| 1 | PubMed/MEDLINE | June 14, 2025 | 99 |
| 2 | EMBASE | June 15, 2025 | 350 |
| 3 | Scopus | June 14, 2025 | 378 |
| 4 | Cochrane Library (CENTRAL) | June 15, 2025 | 62 |
| 5 | ScienceDirect | June 15, 2025 | 112 |
| 6 | Google Scholar* | June 14, 2025 | 3,587 |
| 7 | ClinicalTrials.gov | June 14, 2025 | 52 |
| 8 | Taylor & Francis Online | June 15, 2025 | 16 |
| 9 | Wiley Online Library | June 15, 2025 | 71 |
|  | **Total records identified** |  | **4,727** |

***Note:** Google Scholar screening methodology detailed above. Approximately 3,587 most relevant records were retrieved and screened, representing the top-ranked results by relevance plus additional grey literature sources.

## eAppendix S1B. Full search strategies (verbatim)

### 1. PubMed (NIH)

| Attribute | Details |
| --- | --- |
| Database | PubMed (U.S. National Library of Medicine) |
| Interface | https://pubmed.ncbi.nlm.nih.gov/ |
| Date Searched | June 14, 2025 |
| Records Retrieved | **99** |

Query (advanced search):

((cytomegalovirus OR cytomegalo OR CMV OR HCMV OR "congenital cytomegalovirus" OR "human cytomegalovirus" OR "human herpesvirus 5" OR HHV-5 OR "HHV 5" OR HHV5) AND (immunoglobulin OR immunoglobulins OR hyperimmunoglobulin OR hyperimmunoglobulins OR "hyperimmune globulin" OR HIG OR "Cytomegalovirus-specific hyperimmune globulin") AND (pregnancy OR pregnant OR maternal OR mother OR mothers) AND (prevention OR prevent OR prophylaxis OR transmission OR "vertical transmission" OR "maternal-fetal transmission" OR "congenital infection" OR "passive immunization"))

Filters Applied:

- **Publication Date:** 2015/01/01 – 2025/06/14
- **Species:** Humans
- **Language:** English, Polish
- **Article Types:** Clinical Trial, Randomized Controlled Trial, Controlled Clinical Trial, Observational Study, Cohort Study, Case-Control Study.

### 2. Embase (Embase.com, Elsevier)

| Attribute | Details |
| --- | --- |
| Database | Embase (Elsevier) |
| Interface | www.embase.com |
| Date Searched | June 15, 2025 |
| Records Retrieved | **350** |

Query (advanced search):

('cytomegalovirus'/exp OR cytomegalovirus:ti,ab,kw OR cytomegalo:ti,ab,kw OR cmv:ti,ab,kw OR hcmv:ti,ab,kw OR 'human cytomegalovirus':ti,ab,kw OR 'congenital cytomegalovirus':ti,ab,kw OR 'human herpesvirus 5':ti,ab,kw OR hhv-5:ti,ab,kw OR 'hhv 5':ti,ab,kw OR hhv5:ti,ab,kw) AND ('immunoglobulin'/exp OR 'hyperimmune globulin'/exp OR immunoglobulin*:ti,ab,kw OR hyperimmunoglobulin*:ti,ab,kw OR 'hyperimmune globulin':ti,ab,kw OR hig:ti,ab,kw OR 'cytomegalovirus-specific hyperimmune globulin':ti,ab,kw OR 'intravenous immunoglobulin':ti,ab,kw OR ivig:ti,ab,kw OR 'cmv immunoglobulin':ti,ab,kw) AND ('pregnancy'/exp OR 'pregnant woman'/exp OR 'mother'/exp OR pregnancy:ti,ab,kw OR pregnant:ti,ab,kw OR maternal:ti,ab,kw OR mother*:ti,ab,kw OR 'prenatal care'/exp OR prenatal:ti,ab,kw) AND ('vertical transmission'/exp OR 'mother to child transmission'/exp OR 'fetal infection'/exp OR 'congenital infection'/exp OR 'prophylaxis'/exp OR 'immunization passive'/exp OR prevention:ti,ab,kw OR prophylaxis:ti,ab,kw OR transmission:ti,ab,kw OR 'vertical transmission':ti,ab,kw OR 'maternal-fetal transmission':ti,ab,kw OR 'congenital infection':ti,ab,kw OR 'intrauterine infection':ti,ab,kw)AND [2015-2025]/py

Filters Applied:

- **Publication Year:** 2015–2025
- **Database Subset:** EMBASE (Elsevier)
- **Human Studies Only**

Search Notes:

Search strategy was refined through iterative optimization. Additional synonyms (ivig, prenatal, disease prevention MeSH) were incorporated to maximize sensitivity, increasing yield from initial 47 to final 350 records.

### 3. Scopus (Elsevier)

| Attribute | Details |
| --- | --- |
| Database | Scopus (Elsevier) |
| Interface | www.scopus.com |
| Date Searched | June 14, 2025 |
| Records Retrieved | **378** |

Query (advanced search):

TITLE-ABS-KEY (("cytomegalovirus" OR cytomegalo OR cmv OR hcmv OR "congenital cytomegalovirus" OR "human cytomegalovirus" OR "human herpesvirus 5" OR hhv-5 OR "HHV 5" OR hhv5) AND (immunoglobulin OR immunoglobulins OR hyperimmunoglobulin OR hyperimmunoglobulins OR "hyperimmune globulin" OR hig OR "Cytomegalovirus-specific hyperimmune globulin") AND (pregnancy OR pregnant OR maternal OR mother OR mothers) AND (prevention OR prophylaxis OR transmission OR "vertical transmission" OR "maternal-fetal transmission" OR "congenital infection")

Filters Applied:

- **Document Type:** Article, Conference Paper, Review
- **Publication Year:** 2015–2025
- **Language:** English
- **Subject Area:** Medicine, Immunology and Microbiology, Biochemistry, Genetics and Molecular Biology

### 4. Cochrane Library

| Attribute | Details |
| --- | --- |
| Database | Cochrane Central Register of Controlled Trials |
| Interface | www.cochranelibrary.com |
| Date Searched | June 15, 2025 |
| Records Retrieved | **62** |

Query (advanced search):

([ti,ab,kw]:cytomegalovirus OR [ti,ab,kw]:cmv OR [ti,ab,kw]:"human cytomegalovirus" OR [ti,ab,kw]:"congenital cytomegalovirus" OR [ti,ab,kw]:"human herpesvirus 5" OR [ti,ab,kw]:hhv-5)AND ([ti,ab,kw]:immunoglobulin OR [ti,ab,kw]:immunoglobulins OR [ti,ab,kw]:"hyperimmune globulin" OR [ti,ab,kw]:hig OR [ti,ab,kw]:"cytomegalovirus-specific hyperimmune globulin") AND ([ti,ab,kw]:pregnancy OR [ti,ab,kw]:pregnant OR [ti,ab,kw]:maternal)

**Limits:** Year: 2015–2025; Trials only (CENTRAL).

Filters Applied:

- **Publication Year:** 2015–2025
- **Study Type:** Trials (CENTRAL registry includes only controlled trials)

### 5. ScienceDirect

| Attribute | Details |
| --- | --- |
| Database | ScienceDirect (Elsevier) |
| Interface | www.sciencedirect.com |
| Date Searched | June 15, 2025 |
| Records Retrieved | **112** |

Query (advanced search):

ALL("cytomegalovirus" OR "cmv") AND ALL(immunoglobulin* OR "hyperimmune globulin") AND ALL(pregnancy OR "pregnant women") AND ALL(prevention OR "vertical transmission")

Filters Applied:

- **Publication Date:** 2015–2025
- **Content Type:** Research articles, Review articles
- **Subject Areas:** Medicine and Dentistry, Immunology and Microbiology, Biochemistry, Genetics and Molecular Biology

### 6. Google Scholar

| Attribute | Details |
| --- | --- |
| Database | Google Scholar |
| Interface | scholar.google.com |
| Software | Publish or Perish v8.18.5090.9296 |
| Date Searched | June 14, 2025 |
| Total Results Identified | ~10,000+ (limited by Google Scholar API) |
| Records Retrieved | **3,587** |

Query (as entered in PoP “All of the words / with phrase” boxes):

Title words: cytomegalovirus OR CMV

Keywords: immunoglobulin OR "hyperimmune globulin" OR HIG

Any of the words: pregnancy OR "pregnant women" OR congenital

Exclude words: vaccine OR vaccination

Year range: 2015–2025

Search Settings:

- **Publication Years:** 2015–2025
- **Sort Order:** By relevance (default Google Scholar ranking)
- **Export Method:** Publish or Perish software (batch retrieval)

**Screening Methodology**

Google Scholar identified >10 000 potentially relevant records; due to platform limitations, a two-stage pragmatic approach was implemented to balance breadth and feasibility.

**Stage 1 - Systematic Retrieval (First ≈ 600 results by relevance):**

- Google Scholar’s ranking prioritizes recent and highly-cited literature.
- Publish or Perish software extracted the top ≈ 600 records sorted by relevance and citation metrics.
- This approach aligns with Cochrane Handbook guidance to screen the first 200–500 Google Scholar results for grey literature.

**Stage 2 - Targeted Grey Literature Searching:**

- Forward and backward citation chasing from key included studies.
- Targeted searches for theses, dissertations, and preprints from identified research groups.
- Retrieved approximately 2,987 additional records through these methods

**Rapid Triage Process:**

All 3 587 records underwent dual-reviewer triage to remove clearly ineligible items.

| Exclusion Category | Approximate Count | Criteria |
| --- | --- | --- |
| Wrong study design | ~1,850 | In-vitro/animal studies, non-controlled case reports, editorials/commentaries |
| Wrong outcome | ~900 | Diagnostic or treatment of established infection, non-pregnant populations |
| Wrong intervention | ~450 | Antivirals, vaccines, non-immunoglobulin therapies |
| Non-English | ~230 | Papers without English full text or abstract (e.g., Russian, Chinese, Japanese) |
| Subtotal excluded | **3,430** |  |
| Advanced to formal screening | **157** |  |

Search Notes:

Google Scholar results included journal articles, dissertations, preprints, conference proceedings, and other grey literature not indexed elsewhere. Publish or Perish facilitated transparent documentation of retrieval and export, addressing Google Scholar’s export limitations.

### 7. ClinicalTrials.gov

| Attribute | Details |
| --- | --- |
| Database | ClinicalTrials.gov (U.S. National Library of Medicine) |
| Interface | www.clinicaltrials.gov |
| Date Searched | June 14, 2025 |
| Records Retrieved | **52** |

Query (advanced search):

(cytomegalovirus OR CMV OR Cytomegalo OR HCMV) AND (hyperimmunoglobulin OR hyperimmunoglobulins OR "hyperimmune globulin" OR immunoglobulin OR immunoglobulins OR HIG) AND (pregnancy OR "pregnant women" OR congenital) AND (prevention OR treatment OR trial OR transmission)

Filters Applied:

- **Study Type:** All Studies (Interventional and Observational)
- **Study Start Date:** 01/01/2015 to 06/14/2025
- **Study Results:** All studies (with posted results)
- **Recruitment Status:** Completed

Search Notes:

Limited to completed trials with posted results to capture unpublished outcome data while focusing on studies with available evidence for meta-analysis.

### 8. Taylor & Francis Online

| Attribute | Details |
| --- | --- |
| Database | Taylor & Francis Online |
| Interface | www.tandfonline.com |
| Date Searched | June 15, 2025 |
| Records Retrieved | **16** |

Query (advanced search):

Title: (cytomegalovirus OR CMV)

Abstract: (immunoglobulin OR "hyperimmune globulin" OR HIG)

AND Year: 2015–2025

Filters Applied:

- **Publication Date:** 2015–2025
- **Content Type:** Research Articles, Review Articles
- **Subject:** Medicine, Health & Social Care, Bioscience

### 9. Wiley Online Library

| Attribute | Details |
| --- | --- |
| Database | Wiley Online Library |
| Interface | onlinelibrary.wiley.com |
| Date Searched | June 15, 2025 |
| Records Retrieved | **71** |

Query (advanced search):

Title: (cytomegalovirus OR CMV)

Abstract: (immunoglobulin OR "hyperimmune globulin" OR HIG)

AND Year: 2015–2025

Filters Applied:

- **Publication Date:** 2015–2025
- **Content Type:** Research Articles, Reviews, Clinical Trials
- **Subject:** Medicine, Immunology, Life Sciences

## eAppendix S1C. Deduplication and screening workflow

Deduplication (Zotero 7.0)

All 4 727 records were imported into Zotero 7.0 for automatic and manual duplicate detection (fields: title, author, year, DOI, PMID/PMCID). Duplicates removed: 82, which resulted in 4,645 unique records retained.

Pre-Screening Triage

Of 4,645 unique records, 4,188 were excluded as clearly irrelevant (wrong design, outcome, intervention, or language). This step followed *PRISMA 2020* Item 16a, which permits pre-screen removal of obviously ineligible records. Two reviewers applied the triage independently; disagreements were resolved by consensus.

Title and abstract screening

457 records proceeded to title/abstract screening in Covidence; 431 were excluded, leaving 26 for full-text review.

**Full-Text Assessment**

All 26 reports were retrieved (100 %). Thirteen were excluded (see Supplement 2) for ineligible design (*n* = 10), outcome (*n* = 1), intervention (*n* = 1), or language (*n* = 1).

**Included in the systematic review:** 13 studies (6 controlled studies, 7 single-arm observational).

## eAppendix S1D. Search validation

**Sensitivity Check:** All 13 included studies were identified by our search strategy (100% capture rate), confirming adequate sensitivity.

**Citation Searching:** Forward/backward citation searching of key studies (Devlieger 2021, Hughes 2021) identified 0 additional eligible studies.

**Expert Consultation:** Author contact was not undertaken; no unpublished studies were identified through other means.

### Search Peer Review

The search strategy was developed by the review team and reviewed by an information specialist (MRS Sp. z.o.o., Warsaw, Poland).
