# Supplementary material for: Efficacy of Immunoglobulin Therapy for Secondary Prevention of Congenital Cytomegalovirus Infection: A Systematic Review and Meta-Analysis
Source: Open Forum Infect Dis. 2026 Jul 16;13(7):ofag431. doi: 10.1093/ofid/ofag431 (PMC13397120; doi:10.1093/ofid/ofag431)
Supplement: ofag431_Supplementary_Data [file ofag431_supplementary_data.zip › Supplement_2_Excluded_Studies_rev_2026-06-29.docx]

# Supplement 2. Excluded Studies at Full-Text Assessment

Twenty-six full-text reports were assessed for eligibility. Thirteen were excluded for the following primary reasons: ineligible study design (*n* = 10), wrong outcome (*n* = 1), wrong intervention (n = 1), and unavailable English full text (*n* = 1).

## eTable S2A. Characteristics of Excluded Full-Text Studies (Summary)

| No. | Study (First Author, Year) | Reason for Exclusion | Specific Rationale |
| --- | --- | --- | --- |
| 1 | Aiba, 2017 | Ineligible design | *In vitro* mechanistic study; no pregnant population; no clinical effectiveness outcomes. |
| 2 | Coste Mazeau, 2022 | Ineligible design | *In vitro/ex vivo* placental model; not a clinical comparative effectiveness study. |
| 3 | De la Calle, 2018 | Ineligible design | Single case report; no comparator; not eligible by design criteria. |
| 4 | Gabrielli, 2018 | Ineligible design | Placental histology comparison; lacks eligible fetal/neonatal infection outcomes. |
| 5 | Kagan, 2017 | Ineligible design | Conference abstract only; insufficient methodological detail. |
| 6 | Miescher, 2015 | Ineligible design | Laboratory comparison of HIG vs IVIG; not a clinical intervention in pregnancy. |
| 7 | Nigro, 2023 | Ineligible design | Narrative review and case series; not a primary comparative study. |
| 8 | Penka, 2018 | Ineligible design | Diagnostic-accuracy focus; not aligned with comparative effectiveness outcome. |
| 9 | Saade, 2020 | Ineligible design | Conference abstract; insufficient detail for extraction and bias assessment. |
| 10 | Schampera, 2017 | Ineligible design | *In vitro* neutralization capacity; not a clinical study in pregnant patients. |
| 11 | Rouse, 2025 | Wrong outcome | Maternal/obstetric outcomes only; no fetal/neonatal infection or transmission. |
| 12 | De la Calle, 2022 | Wrong intervention | Combined treatment with valaciclovir and immunoglobulin; no HIG-only arm. |
| 13 | Rogozina, 2019 | Full text not available in English | Report available only in Russian; English translation unavailable for assessment. |

## eAppendix S2B. Detailed exclusion rationales

### Studies excluded due to wrong study design (*n* = 10)

**1. Aiba, 2017**

- **Citation:** **Aiba N, Shiraki A, Yajima M, Oyama Y, Yoshida Y, et al.** Interaction of immunoglobulin with cytomegalovirus-infected cells. *Viral Immunology.* 2017. doi:10.1089/vim.2016.0151
- **Reason:** Ineligible design
- **Rationale:** This *in vitro* mechanistic study investigated the interaction between IVIG and CMV-infected cells. The design was laboratory-based, assessing viral protein synthesis and antibody half-life without a clinical or pregnant population. No vertical transmission outcomes were reported.

**2. Coste Mazeau, 2022**

- **Coste Mazeau P, Jacquet C, Muller C, et al.** Potential of Anti-CMV Immunoglobulin Cytotect CP® in vitro and ex vivo in a first-trimester placenta model. *Microorganisms.* 2022;10(4):694. doi:10.3390/microorganisms10040694
- **Reason:** Ineligible design
- **Rationale:** This study employed *in vitro* neutralization assays and *ex vivo* assays using a **first-trimester placenta model** to characterize the potential of hyperimmune globulin Cytotect CP®. The design uses non-clinical models and therefore is ineligible for human systematic review assessing clinical effectiveness

**3. De la Calle, 2018**

- **De la Calle M, Baquero F, Rodriguez R, et al.** Successful treatment of intrauterine CMV infection with an intraventricular cyst in a dichorionic diamniotic twin gestation using CMV immunoglobulin. *J Matern Fetal Neonatal Med.* 2018;31(16):2226-2229. doi:10.1080/14767058.2017.1338259
- **Reason:** Ineligible design
- **Rationale:** This was excluded as a **case report** detailing the successful treatment of intrauterine CMV infection in a dichorionic diamniotic twin gestation using CMV immunoglobulin (CMVIG). Case reports do not constitute robust evidence for systematic reviews focused on study design integrity.

**4. Gabrielli, 2018**

- **Gabrielli L, Bonasoni MP, Foschini MP, et al.** Histological analysis of term placentas from HIG-treated and untreated mothers with primary CMV infection. *Fetal Diagn Ther.* 2018;45(2):111-117. doi:10.1159/000487302
- **Reason:** Ineligible design
- **Rationale:** This publication represents a **secondary analysis** focused on the Histological Analysis of Term Placentas collected for the CHIP study. While the original study was a randomized trial, the histological outcomes were deemed secondary or outside the scope of the primary clinical efficacy review.

**5. Kagan, 2017**

- **Kagan KO, Hoopmann M, …** EP17.03: Effectiveness of a 2-weekly HIG protocol in first-trimester CMV infection. *Ultrasound in Obstet Gynecol.* 2017. doi:10.1002/uog.18596
- **Reason:** Ineligible design
- **Rationale: Conference abstract** only; insufficient methodological detail. The design is a small pilot study, lacking the necessary control or randomization for high-level inclusion. This publication describes an **observational study** of 20 pregnant women to examine the effectiveness of a biweekly HIG protocol.

**6. Miescher, 2015**

- **Miescher SM, Huber TM, Kühne M, Lieby P, …** In vitro evaluation of CMV-specific hyperimmune globulins vs standard IVIG. *Vox Sanguinis.* 2015. doi:10.1111/vox.12246
- **Reason:** Ineligible design
- **Rationale:** Laboratory comparison of HIG and IVIG neutralizing titers and ELISA activity; no clinical population or outcomes..

**7. Nigro, 2023**

- **Nigro G, Muselli M,** On Behalf Of The Congenital Cytomegalic Disease Collaborating Group null. Prevention of Congenital Cytomegalovirus Infection: Review and Case Series of Valaciclovir versus Hyperimmune Globulin Therapy. *Viruses*. 2023;15(6):1376. doi:[10.3390/v15061376](https://doi.org/10.3390/v15061376)
- **Reason:** Ineligible design
- **Rationale:** Narrative review and case series reporting previously published cases; not a primary comparative study and not eligible for data extraction.

**8. Penka, 2018**

- **Penka L, Kagan KO, Goelz R, Hamprecht K**. Comparison of quantitative real-time PCR and short-term (18-hour) microculture in diagnosis of fetal cytomegalovirus infection: Impact of hyperimmunoglobulin treatment. *Prenat Diagn*. 2018;38(12):936-942. doi:[10.1002/pd.5338](https://doi.org/10.1002/pd.5338)
- **Reason:** Ineligible design
- **Rationale: diagnostic accuracy** focus; not a comparative clinical effectiveness design for transmission outcome; This study was excluded as its primary design focused on a **retrospective diagnostic study** comparing the analytical performance of quantitative real-time PCR (rtPCR) and quantitative short-term microculture from amniotic fluid samples; While it addresses the impact of HIG treatment, its design centers on laboratory/diagnostic method correlation rather than clinical outcomes.

**9. Saade, 2020**

- **Saade GR.** The effect of treatment of maternal CMV infection on development of placental syndrome. *Am J Obstet Gynecol.* 2020; 222(1):S2-S3. doi:10.1016/j.ajog.2019.11.018
- **Reason:** Ineligible design
- **Rationale:** Conference abstract; insufficient methodological detail for extraction and risk-of-bias assessment. The report summarizes a secondary analysis of a multicenter randomized trial assessing the potential effect of HIG on placental outcomes.

**10. Schampera, 2017**

- **Schampera MS, Schweinzer K, Abele H, et al**. Comparison of cytomegalovirus (CMV)-specific neutralization capacity of hyperimmunoglobulin (HIG) versus standard intravenous immunoglobulin (IVIG) preparations: Impact of CMV IgG normalization. *Journal of Clinical Virology*. 2017;90:40-45. doi:[10.1016/j.jcv.2017.03.005](https://doi.org/10.1016/j.jcv.2017.03.005)
- **Reason:** Ineligible design
- **Rationale:** *In vitro* laboratory comparison of CMV-specific neutralization capacity between HIG and standard IVIG preparations using microneutralization protocols; no clinical data.

### Study excluded due to wrong outcome (*n* = 1)

**11. Rouse, 2025**

- **Rouse DJ, MacPherson C, Saade GR, et al.** The association of CMV hyperimmune globulin with adverse pregnancy outcomes. *Am J Obstet Gynecol.* 2025;0(0). doi:10.1016/j.ajog.2025.04.014
- **Reason:** Wrong outcome
- **Rationale:** This publication’s primary focus was assessing whether maternal receipt of CMV HIG was significantly associated with a **composite of adverse pregnancy outcomes** (GHTN, preeclampsia, SGA, placental abruption, PTD, or perinatal death), rather than focusing on the prevention of congenital CMV infection itself; the primary composite outcome for this secondary analysis was explicitly chosen to encompass adverse pregnancy outcomes, defining the wrong outcome for the review

### Study excluded due to wrong intervention (*n* = 1)

**12. De La Calle, 2022**

- **De la Calle M, Baquero-Artigao F, Rodríguez-Molino P, et al.** Combined treatment with immunoglobulin and valaciclovir in pregnant women with CMV infection and high risk of symptomatic fetal disease. *J Matern Fetal Neonatal Med.* 2022;35(16):3196-3200. doi:10.1080/14767058.2020.1815188
- **Reason:** Wrong intervention
- **Rationale:** This publication investigates a **combined** treatment with **immunoglobulin and valaciclovir;** since the focus was on the simultaneous use of both drugs, it represents an ineligible mixed intervention if the review mandates CMV-HIG monotherapy studies.

### Study excluded due to non-english full text (*n* = 1)

**13. Rogozina, 2019**

- **Rogozina NV, Vasilev VV, Grineva AA, et al.** Анте- и постнатальная диагностика и комплексное лечение врожденной цитомегаловирусной инфекции [Ante- and postnatal diagnostics and complex treatment of congenital CMV infection]. *Rossiyskiy Vestnik Perinatologii i Pediatrii.* 2019;64(6):89-93. doi:10.21508/1027-4065-2019-64-6-89-93
- **Reason:** Full text not available in English
- **Rationale:** This publication was excluded because the full text, written in Russian, contains essential case details and discussion unavailable in English. The English abstract suggested potential eligibility, but full-text translation was not feasible, and information was insufficient for data extraction.
