# Supplementary material for: Efficacy of Immunoglobulin Therapy for Secondary Prevention of Congenital Cytomegalovirus Infection: A Systematic Review and Meta-Analysis
Source: Open Forum Infect Dis. 2026 Jul 16;13(7):ofag431. doi: 10.1093/ofid/ofag431 (PMC13397120; doi:10.1093/ofid/ofag431)
Supplement: ofag431_Supplementary_Data [file ofag431_supplementary_data.zip › Supplement_3_GRADE_Evidence_Profile_rev_2026-06-29.docx]

# Supplement 3. GRADE Evidence Profile

## Methods for GRADE Assessment

Certainty of evidence was evaluated for each critical outcome using the Grading of Recommendations, Assessment, Development and Evaluation (GRADE) approach. Two reviewers (KG,TI) independently assessed five domains: risk of bias, inconsistency, indirectness, imprecision, and publication bias, following the Cochrane Handbook for Systematic Reviews of Interventions (version 6.5, Chapter 14). Disagreements were resolved through discussion with a third reviewer (AKC). We rated the certainty of evidence for each outcome as high, moderate, low, or very low.

Evidence from randomized controlled trials started at high certainty; evidence from nonrandomized studies started at low certainty. Certainty could be downgraded for serious or very serious limitations in any of the five domains. Conversely, certainty could be upgraded for a large magnitude of effect, presence of a dose-response gradient, or if residual confounding would reduce rather than create the observed effect.

All GRADE assessments refer to the critical outcome of congenital CMV transmission (vertical transmission from mother to fetus/neonate), with an additional assessment provided for the pooled transmission rate in HIG-treated pregnancies (non-comparative, observational estimate).

## eTable S3A. Summary of Findings (GRADE)

| **Outcome** | **Studies (design)** | **Participants (*n*)** | **Assumed risk**^*^ | **Corresponding risk**^†^ | **Relative effect (95% CI)** | **Certainty (GRADE)** | **Interpretation** |
| --- | --- | --- | --- | --- | --- | --- | --- |
| Congenital CMV transmission (HIG vs control) | 6 controlled studies (2 RCTs, 4 nonrandomized cohorts with comparators) | 1,177 (578 HIG, 599 control) | 200 per 1000 (20.0%) | 146 per 1000 (108-200) [14.6% (10.8%-20.0%)] | RR 0.73 (0.54-1.00);  p = .051 | ⊕⊕◯◯ **LOW** | HIG *may* reduce congenital CMV transmission by about 54 cases per 1000 pregnancies, but the CI includes no effect. Evidence downgraded for **risk of bias** (serious ROBINS-I ratings in cohort studies and one RoB 2 high-risk trial) and **imprecision** (borderline CI). |
| Overall cCMV transmission after HIG (prevalence) | 13 studies (mixed: 6 controlled, 7 single-arm observational) | 1,024 | Not applicable (no control) | 272 per 1000 (188-377) [27.2% (18.8%-37.7%)] | Not applicable (observational pooled proportion) | ⊕◯◯◯ **VERY LOW** | Very uncertain evidence. Non-comparative design, high heterogeneity (I² ≈ 90%), and indirectness for treatment effect. |

***Assumed risk** represents the median baseline transmission rate in control groups from comparative studies (≈20%).

†Corresponding risk calculated by multiplying assumed risk by pooled RR.

**Abbreviations:** CI, confidence interval; CMV, cytomegalovirus; GRADE, Grading of Recommendations Assessment, Development and Evaluation; HIG, hyperimmune globulin; RCT, randomized controlled trial; RR, risk ratio.

## eTable S3B. Detailed Evidence Profile by GRADE Domain

| **Outcome** | **Risk of Bias** | **Inconsistency** | **Indirectness** | **Imprecision** | **Publication Bias** | **Other Considerations** | **Overall  Certainty** |
| --- | --- | --- | --- | --- | --- | --- | --- |
| **Congenital CMV transmission (HIG vs control)** | **Serious (-1):** Four cohort studies were judged at serious overall risk of bias by ROBINS-I, mainly due to confounding and selection of participants. Of the two randomized trials assessed with RoB 2, Hughes 2021 was low risk across domains, whereas Devlieger 2021 was high risk overall due to deviations from intended interventions (open-label design with off-label HIG use in controls) and substantial differential missing outcome data among seroconverters. | **Not serious (0):** Moderate heterogeneity (I² = 54.5%, 95% CI 0%-82%); sensitivity analysis identified 2 influential studies; heterogeneity largely explained by study design differences. | **Not serious (0):** Population, intervention, comparator, and outcome directly address research question (PICO fully aligned). | **Serious (-1):** CI crosses RR = 1.0 (includes both benefit and no effect);  p = .051; wide prediction interval (-0.88 to 0.26). | **Not detected (0):** Egger's test p = .943; funnel plot symmetric; weight-function model consistent with primary estimate. Small number of studies limits power of tests. | **No upgrade:** No large effect (RR not <0.5), no clear dose-response, residual confounding likely inflates rather than reduces observed effect. | **⊕⊕◯◯ LOW** (started HIGH for RCTs/MODERATE for mixed evidence; downgraded twice) |
| **Pooled transmission rate in HIG-treated pregnancies** | **Serious (-1):** All studies observational; no random allocation; potential selection bias (sicker patients may receive HIG); measurement variability. | **Very serious (-2):** Extremely high heterogeneity (I² = 90.1%, 95% CI 85%-94%); study estimates ranged 6.5%-63.6%; not fully explained by subgroups. | **Serious (-1):** Non-comparative design; pooled proportion cannot directly inform treatment efficacy; indirect for primary research question. | **Serious (-1):** Wide confidence interval (18.8%-37.7%); small sample sizes in many studies. | **Not detected (0):** Egger's test p = .287; however, high heterogeneity limits interpretation. | Not applicable (observational pooled estimate). | **⊕◯◯◯ VERY LOW** (started LOW; downgraded 3 additional times) |

## eAppendix S3C. Narrative Rationale for GRADE Judgments

### Primary Outcome: Congenital CMV Transmission (HIG vs Control)

**Evidence Summary:**

Six controlled studies (2 RCTs: Devlieger 2021, Hughes 2021; 4 nonrandomized cohorts with control groups: Nigro 2015, Nigro 2020, Richtmann 2022, Seidel 2020) including 1,177 participants (578 HIG, 599 control) were meta-analyzed. The pooled risk ratio was 0.73 (95% CI 0.54-1.00, p = .051), suggesting a possible 27% relative reduction in congenital CMV transmission, but the confidence interval includes no effect (RR = 1.0).

**GRADE Domain Assessments:**

**Risk of Bias (-1 level):**

Serious limitations identified. Four nonrandomized cohort studies were rated as having serious overall risk of bias using the ROBINS-I tool, primarily due to:

- Confounding by indication (women receiving HIG may differ systematically from controls)
- Selection bias (non-random allocation to intervention)
- Measurement bias (variable outcome ascertainment methods)

Of the two randomized trials assessed with RoB 2, Hughes 2021 was low risk across domains, whereas Devlieger 2021 was high risk overall due to deviations from intended interventions (open-label design with off-label HIG use in controls) and substantial differential missing outcome data among seroconverters. Overall, the controlled evidence was downgraded by one level for risk of bias.

- Devlieger 2021: Open-label design; differential attrition in control group
- Hughes 2021: Large proportion of missing outcome data (>20%)

**Inconsistency (0 levels):**

Not downgraded. Heterogeneity was moderate (I² = 54.5%, 95% CI 0%-82%, τ² = 0.035, Q = 11.00, p = .051). Leave-one-out sensitivity analysis identified two influential studies (Hughes 2021, Nigro 2020); excluding both reduced I² to 0% but removed 58% of participants without methodological justification. Heterogeneity is largely explained by differences in study design (RCT vs cohort), timing of intervention, and HIG dosing protocols.

**Indirectness (0 levels):**

Not downgraded. The population (pregnant women with primary CMV infection), intervention (CMV-specific hyperimmune globulin), comparator (placebo/usual care/no treatment), and outcome (congenital CMV infection confirmed by PCR/culture within 3 weeks postpartum) directly address the research question. All studies evaluated prevention of vertical transmission, which is the critical patient-important outcome.

**Imprecision (-1 level):**

Serious imprecision. The 95% confidence interval crosses the null value (RR = 1.0), ranging from RR 0.54 (potentially meaningful benefit) to RR 1.00 (no effect). The p-value narrowly missed conventional significance (p = .051). The prediction interval for future studies was wide (-0.88 to 0.26 on the log scale), indicating substantial uncertainty about the effect in new populations. The optimal information size criterion (number of events needed for adequate power) was not met.

**Publication Bias (0 levels):**

Not downgraded. Egger's regression test for funnel plot asymmetry was non-significant (*t* = -0.08, *df* = 4, *p* = .943), and visual inspection showed no obvious asymmetry. Weight-function models adjusting for potential selective reporting yielded estimates nearly identical to the primary analysis (adjusted log RR -0.29, 95% CI -0.58 to 0.00). However, with only 6 studies, statistical tests for publication bias have low power, and the possibility of selective reporting cannot be excluded entirely.

**Other Factors (No Upgrade):**

- **No large effect:** RR = 0.73 does not meet GRADE criteria for large (RR <0.5) or very large (RR <0.2) effects
- **No dose-response:** Insufficient data on HIG dosing and timing to assess gradient
- **Residual confounding:** If present, likely inflates the observed protective effect (women receiving HIG may have better overall care) rather than reducing it, so no upgrade warranted

**Overall Certainty: ⊕⊕◯◯ LOW**

Starting from HIGH certainty for the 2 RCTs and MODERATE for the overall body of mixed evidence, we downgraded twice: once for serious risk of bias and once for serious imprecision. Low certainty indicates that the true effect may be substantially different from the estimated effect.

**Clinical Interpretation:**

There is low-certainty evidence that HIG may reduce congenital CMV transmission. However, the evidence does not definitively establish benefit, and the confidence interval includes the possibility of no effect. Further, high-quality randomized controlled trials are needed before routine clinical use can be recommended.

### Secondary Outcome: Pooled Transmission Rate in HIG-Treated Pregnancies (Non-Comparative)

**Evidence Summary:**

Thirteen studies (all designs, including the 6 controlled studies plus 7 single-arm observational cohorts) reported congenital CMV transmission rates in 1,024 pregnancies receiving HIG. The pooled prevalence was 27.2% (95% CI 18.8%-37.7%), with very high heterogeneity (I² = 90.1%, 95% CI 84.9%-93.5%, τ² = 0.61). Individual study estimates ranged from 6.5% (Kagan 2021) to 63.6% (Nigro 2015).

**Important Limitation:**

This is a **non-comparative, observational estimate** that cannot inform treatment efficacy. It represents the transmission rate observed in HIG-treated pregnancies but does not indicate whether HIG reduced transmission compared to no treatment. This outcome is included for descriptive purposes only and should not be used for clinical decision-making regarding HIG efficacy.

**GRADE Domain Assessments:**

**Risk of Bias (-1 level):**

Serious limitations. All studies were observational (no random allocation). Even the controlled studies contribute observational data to this pooled proportion estimate. Potential biases include:

- Selection bias (indication for HIG use; severity of maternal infection)
- Measurement bias (variable diagnostic methods across studies)
- No adjustment for confounders in single-arm studies

**Inconsistency (-2 levels):**

Very serious inconsistency. Heterogeneity was extremely high (I² = 90.1%), indicating that 90% of observed variation reflects true differences in transmission rates rather than sampling error. Study estimates varied 10-fold (6.5%-63.6%). Heterogeneity was not adequately explained by:

- Timing of HIG administration
- Gestational age at maternal infection
- HIG dosing regimen
- Geographic region

Sensitivity analyses excluding two influential studies (Nigro 2015, Kagan 2021) reduced I² to 36% but excluded 30% of participants without methodological justification.

**Indirectness (-1 level):**

Serious indirectness. The pooled transmission rate in HIG-treated pregnancies does not directly answer the research question ("Does HIG reduce vertical transmission compared to no treatment?"). Without a control group, this estimate cannot inform treatment efficacy. Historical transmission rates (typically 30-40% without treatment) suggest HIG may reduce transmission, but this indirect comparison is insufficient for causal inference.

**Imprecision (-1 level):**

Serious imprecision. The 95% confidence interval is wide (18.8%-37.7%), reflecting both sampling error and true heterogeneity. Many contributing studies had small sample sizes (n <50), limiting precision.

**Publication Bias (0 levels):**

Not downgraded. Egger's test was non-significant (t = -1.12, p = .287), suggesting no strong evidence of funnel plot asymmetry. However, with extremely high heterogeneity, publication bias assessment is challenging and results should be interpreted cautiously.

**Overall Certainty: ⊕◯◯◯ VERY LOW**

Starting from LOW certainty for observational evidence, we downgraded three additional levels (one for risk of bias, two for inconsistency, one for indirectness, one for imprecision = 4 downgrades, capped at VERY LOW). Very low certainty indicates that we have very little confidence in the effect estimate, and the true effect is likely to be substantially different from the estimate.

**Clinical Interpretation:**

The pooled transmission rate of 27.2% in HIG-treated pregnancies cannot inform clinical decision-making about HIG efficacy. This non-comparative estimate is provided for descriptive context only. Clinicians should base decisions on comparative evidence (HIG vs control), which showed low-certainty evidence of possible benefit but with substantial uncertainty.
